# Knowledge Requirements and Unmet Needs of Informal Caregivers of Patients with End-Stage Kidney Disease (ESKD) Receiving Haemodialysis: A Narrative Review

**DOI:** 10.3390/healthcare10010057

**Published:** 2021-12-29

**Authors:** Michael Matthews, Joanne Reid, Clare McKeaveney, Helen Noble

**Affiliations:** Medical Biology Centre, School of Nursing and Midwifery, Queen’s University, Belfast BT9 7BL, UK; j.reid@qub.ac.uk (J.R.); c.mckeaveney@qub.ac.uk (C.M.); helen.noble@qub.ac.uk (H.N.)

**Keywords:** end-stage kidney disease, informal caregivers, unmet needs, supportive interventions, knowledge, skills, narrative review

## Abstract

Background: Patients with end-stage kidney disease receiving haemodialysis rely heavily on informal caregivers to support them living at home. Informal caregiving may exact a toll on caregivers’ physical, emotional, and social well-being, impacting negatively on their overall quality of life. The aim of this narrative review is to report knowledge requirements and needs of informal caregivers of patients with end stage kidney disease (ESKD) receiving haemodialysis. Methods: The review followed the Preferred Reporting Items for Reporting Systematic Reviews and Meta-analyses (PRISMA). Five electronic databases were searched: Web of Science, PsycINFO, Embase, Medline, and CINAHL to identify the experiences and unmet needs of informal caregivers of patients with end stage kidney disease (ESKD) receiving haemodialysis. Results: Eighteen papers were included in the review and incorporated a range of methodological approaches. There are several gaps in the current literature around knowledge and informational needs and skills required by informal caregivers, such as signs and symptoms of potential complications, dietary requirements, and medication management. Although most research studies in this review illustrate the difficulties and challenges faced by informal caregivers, there is a paucity of information as to which support mechanisms would benefit caregivers. Conclusion: Informal caregivers provide invaluable assistance in supporting people with ESKD undergoing haemodialysis. These informal caregivers however experience multiple unmet needs which has a detrimental effect on their health and negatively influences the extent to which they can adequately care for patients. The development of supportive interventions is essential to ensure that informal caregivers have the requisite knowledge and skills to allow them to carry out their vital role.

## 1. Introduction

Chronic kidney disease progresses along a five-stage trajectory. Stage 5 is termed end stage kidney disease (ESKD) [1] and occurs when the glomerular filtration rate of the kidney is less than 15 mL/min, at which point persons require haemodialysis, peritoneal dialysis, or transplantation to sustain life [2]. The prevalence of chronic kidney disease (CKD) is increasing in both developed and developing countries. Worldwide, over 1.5 million individuals receive regular haemodialysis, a number which is projected to double in the next decade [3]. In the United Kingdom (UK) in 2019 there were 7845 adults who commenced renal replacement therapy, a number which is comparable to the previous year. There were 68,111 adult patients receiving renal replacement therapy for ESKD in the UK in 2019 an increase of 2.5% from 2018 [4].

The widespread availability of haemodialysis saves and prolongs the lives of patients with ESKD [5]. These patients however suffer and experience many symptoms and complications, such as profound fatigue, nausea, insomnia, hypotension, and muscle cramp, in addition they are required to adhere to extensive medication regimens, and dietary and fluid restrictions, all of which impact on their ability to travel, fulfil social activities, and sustain employment [6,7], which often translate into a heavy care burden for their informal caregivers [8,9,10]. When a patient with a diagnosis of ESKD commences haemodialysis treatment, life changes not only for the patient, but also for those who are emotionally and practically involved in supporting and providing care for these patients [5]. Given the complexities of ESKD and haemodialysis, patients increasingly rely on informal carers to help manage this debilitating condition and support them in their everyday lives [11]. Informal caregivers are often family members, close friends or neighbours who voluntarily provide practical care and emotional support for their loved ones when they require haemodialysis [12,13]. Studies have shown that informal caregivers are challenged in caring for patients receiving haemodialysis, and in doing so experience considerable physical and psychological pressures [14,15]. One key contributing factor to informal caregiver burden emerging as a considerable concern in renal healthcare is the increasing numbers of older people with multiple co-morbidities receiving haemodialysis [16]. In the United Kingdom (UK) patients aged over 65 years represent the fastest growing group of the dialysis population [17]. As a result of the challenges faced by informal caregivers of patients with ESKD receiving haemodialysis, they may be fearful, feel vulnerable, isolated, experience conflict with their other roles or responsibilities, and feel overwhelmed by their responsibilities [18,19,20]. In addition, informal caregivers convey uncertainty about their role, encounter difficulties in accessing the healthcare system, lack treatment-related and disease-related knowledge and report unmet support needs [21].

Informal caregivers of patients with ESKD receiving haemodialysis have consistently received little attention in both research and practice [12,22], and lack both support mechanisms and the knowledge to enable them to carry out their caring duties effectively [3]. As informal carers play a crucial role in the daily management of patients with ESKD receiving haemodialysis there is a need to identify their needs and knowledge requirements in relation to their caring role, so that educative and supportive interventions can be developed which both recognise and respond to these needs. This narrative review aims to report knowledge requirements and needs of informal carers of patients with ESKD receiving haemodialysis. The aim of this review was to ascertain informal caregivers’ knowledge regarding the care they provide, explore how knowledge has impacted on informal caregivers’ ability when providing care to patients with ESKD receiving haemodialysis and to identify the needs and experiences of informal caregivers of patients with ESKD receiving haemodialysis.

## 2. Materials and Methods

### 2.1. Search Strategy 

**Figure 1:**
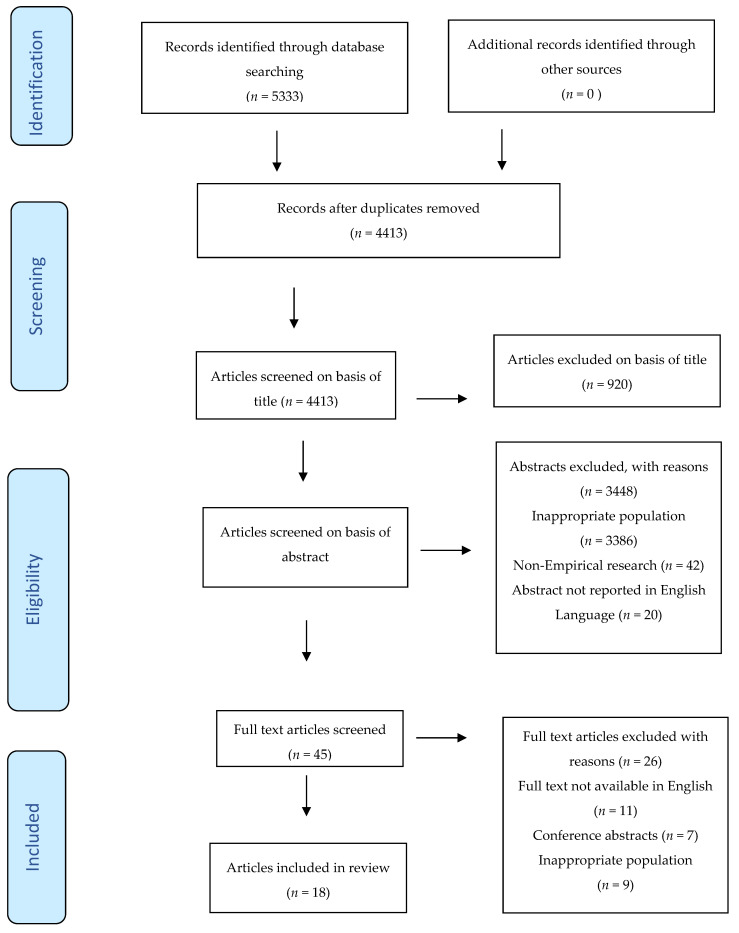
PRISMA flowchart diagram.

This narrative review adhered to the Preferred Reporting Items for Reporting Systematic Reviews and Meta-analyses (PRISMA) guidelines [23] which was used to depict the flow of information through the different phases of the review [24], as illustrated in flow diagram (Figure 1). PRISMA guidelines improves the quality and transparency of the data included [23]. A narrative review which is a scholarly summary was chosen as it offers a breadth of literature coverage, provides interpretation and critique thus eliciting a deeper understanding of a certain phenomenon [25,26]. 

A systematic search using the search terms and index terms related to informal carers was conducted. Two different methods were used to search for appropriate literature: database searching and citation searching. Following consultation with a subject librarian five electronic databases (Web of Science, PsycINFO, Embase, Medline and CINAHL) were searched. The purpose of the search was to identify relevant publications that had reported on the knowledge, needs and experience of informal caregivers of people with ESKD receiving haemodialysis. The search was limited to English language studies published between 1 February 2010 until 1 February 2020, as we wanted to build our review on more recent literature to reflect the difficulties and challenges associated with providing informal care to patients with ESKD on haemodialysis. This is emerging as a major issue in renal healthcare due to the increasing numbers of older people with multiple comorbidities being accepted unto haemodialysis. An example of the search strategy used for the Medline database is shown in Table 1.

### 2.2. Inclusion and Exclusion Criteria 

The inclusion and exclusion criteria were as follows: 

Inclusion Criteria

- Empirical articles, including both qualitative and quantitative studies
- Patients with a diagnosis of ESKD
- Patients receiving hospital-based haemodialysis
- Studies conducted on participants over the age of 18 years
- Studies reporting knowledge and needs of informal caregivers
- Studies reported in English Language only

Exclusion Criteria

- Editorial, theoretical, discussion or news articles, conference abstracts or dissertations
- Patients receiving home-based haemodialysis, peritoneal dialysis of having received a transplant
- Studies published more than 10 years ago.

### 2.3. Data Extraction and Synthesis 

A narrative synthesis was conducted which focused on identifying themes relating to knowledge and needs of informal carers. Following this, a data extraction table (Cf. Table S1) was developed to identify the necessary information to address the aim and objectives of the review. The relevant articles were then critically appraised using the Critical Appraisal Skills Programme (CASP) to systematically assess the trustworthiness and quality of data contained within the selected studies and to draw conclusive evidence (CASP 2020). Using the CASP rating scores, the quality of articles can be classified as high, moderate, or low [27].

The evidence and information obtained from the relevant studies was gathered and analysed to synthesise commonly occurring themes which included: access to and provision of knowledge and information to assist informal caregivers in providing care, factors associated with psychological wellbeing in informal caregivers of people on haemodialysis and caregiver burden as described by informal caregivers of patients undergoing haemodialysis treatment, which emerged from the literature search. Within each one of the themes there were related sub-themes. 

## 3. Results

### 3.1. Characteristics of Included Studies 

The characteristics of the final studies are shown in Table S1. In total 18 papers which reported empirical findings relating to knowledge, needs, and ability of informal carers to care for patients with ESKD receiving haemodialysis were included in this narrative review. Seven studies took place In Iran [3,8,28,29,30,31,32], whilst the remaining eleven studies were based on populations in Vietnam [16], China [33,34,35], India [36,37], Australia [38], Canada [39], United States of America [40], Jordan [41], and Turkey [41]. 

The included studies used a range of methodological approaches including interventional studies [29,35], qualitative studies [3,8,30,31,38], and quantitative studies [33,34,36,41]. The remaining studies were descriptive/analytical [28,37,40,41], a non-experimental cross-sectional study [16], correlational study [32], and secondary analysis of a survey [39]. Participant recruitment in the studies varied between 30 and 150 participants. The 18 studies included informal caregivers (*n* = 1927), patients (*n* = 145) and healthcare professionals (*n* = 521). The CASP ratings are included within the data extraction table. Five papers were classified as moderate quality and 13 of low quality.

### 3.2. Theme 1—Provision of Information to Assist Informal Caregivers in Providing Care

This theme synthesised the knowledge and information support needs of informal carers of patients receiving haemodialysis. 

#### 3.2.1. Sub-Theme 1—Informal Caregivers’ Access to and Provision of Sources of Knowledge to Assist in the Management of Patient Symptoms

At the initiation of haemodialysis treatment, general information about caring for the patient undergoing haemodialysis was generally provided by doctors, supported by some nursing input, however no standardised approach was outlined [8,37]. Informal carers identified nurses as pivotal in accessing information relating to immediate concerns such as medications and dietary and fluid requirements [8,37]. Informal carers identified an unmet need relating to the pathology of kidney failure, signs and symptoms of potential complications (lack of energy and puritis), and advice on the practical aspects of caregiving [8,37,39]. Patients relaying medical information to their carers from healthcare providers was problematic [8,37], as missing or inaccurate information was commonly reported. A lack of information led to informal caregivers having feelings of inability to cope and manage complications experienced by patients [8,37]. 

#### 3.2.2. Sub-Theme 2—Skills Provision for Informal Carers through Learning Strategies

Informal caregivers used resources such as books and the internet in the absence of information provided by healthcare professionals [8]. This offered informal caregivers a limited understanding of the disease process, specific care needs, and potential complications which might occur, and therefore did not really increase their ability to deliver effective care [3]. This experiential learning was not found useful as informal carers often faced a plethora of information, the majority of which was for general ill-health and non-specific to caring for patients with ESKD receiving haemodialysis [37].

Only two educational interventions were identified, with both studies seeking to examine the effectiveness of problem-focused coping strategies, such as communication skills, anger management, and deep breathing [29], and the education of caregivers on a range of core areas. These core areas related to the care of patients undergoing haemodialysis such as diet and nutrition, blood pressure monitoring, treating potential complications and available support services [35] on caregiving outcome scores. Both interventions reported significant differences in caregiving outcome scores before and after the interventions (*p* < 0.001) [29] and (*p* < 0.05) [35].

### 3.3. Theme 2—Psychosocial Factors Associated with Coping in Caregiving

The second theme synthesised psychosocial factors which assist informal caregivers deal and respond to the changing and complex needs of patients with ESKD receiving haemodialysis.

#### 3.3.1. Sub-Theme 1—Positive Factors Associated with Informal Caregiving 

Informal caregivers advocated the need for self-care to help them adjust to their caregiving role. Self-nurturing was a skill used by caregivers to help them adjust to their caregiving role. Caregivers stated that when they took care of themselves, they felt more positive and able to cope more efficiently while still dealing with the challenges and stresses arising from their caregiving situation [3]. They believed that regardless of the demands in caring for haemodialysis patients, supporting their own well-being was essential as failure to do so could lead to stress, anger, and reduced physical and emotional functioning, all of which could have a negative impact upon one’s ability to deliver effective care and could in turn cause the patients they were caring for unnecessary anxiety and stress [3]. 

Informal carers identified peer support as a valuable resource providing them with practical information about kidney disease and haemodialysis treatment [8,39]. Peer supporters offered informal carers empathy and understanding by advising carers on potential strategies and potential solutions to the problems they were experiencing [8]. Such peer support was often informal taking the form of casual conversations in the waiting area while the patient was attending for haemodialysis treatment.

The impact of a religious and/or spiritual dimension as a coping strategy employed by informal caregivers was explored in two studies [14,32]. Both studies emanate from an Islamic context. There was a significant inverse relationship between caregiver burden scores and spirituality, which illustrates that carers with higher spiritual well-being scores expressed less caregiver burden [14,32]. While the limited literature available suggests that spirituality plays an important role in reducing caregiver burden, there is a need for more research to be conducted to explore the relationship between spirituality and caregiver burden in different cultures and religions.

#### 3.3.2. Sub-Theme 2—Relationship between Caregiver Burden, Quality of Life and Depression

Informal caregivers reported that caregiving could be both emotionally and physically challenging and was associated with a decline in the health status and quality of life of the caregiver [8,30,31,38]. The gradual decline in the patient’s condition, and periods of acute illness resulted in uncertainty and led to a decline in caring capacity and contributed to psychological burden and reduction in quality of life (QoL) of informal caregivers [30,31]. Caregivers often felt overwhelmed making medical appointments, arranging medication regimens, and managing finances [14,38]. This could lead to poor coping mechanisms such as overeating, drinking excessive amounts of fizzy drinks, and chain smoking [38]. 

Caregiver burden varied considerably across studies and was associated with a variety of tasks including monitoring symptoms, treatment related tasks, emotional support, and provision of transport to and from the dialysis facility [16,41]. Caregiver burden scale scores were lower in spouses acting as caregivers when compared to other caregivers such as siblings and children (*p* = 0.025) [41]. Spouses with their own health problems acting as informal caregivers reported a significant higher level of burden compared to those who did not have any health problems (*p* < 0.01) [16]. Factors which resulted in lower caregiver burden was the greater capability of the patient to attend to self-care needs (*p* < 0.001) and the absence of other chronic diseases in haemodialysis patients (*p* < 0.001) [28]. Conflicting evidence surrounds caregivers’ level of burden, QoL and their educational achievements. There was a significant relationship between caregivers’ level of education and care burden (*p* < 0.001) meaning that higher educational attainment decreased caregiver burden and improved QoL [28,34]. Evidence contrary to these findings however were identified in that higher educational achievement was associated with lower QoL scores (*p* = 0.31) [40].

A study examining the incidence and degree of depression, marital dissatisfaction, and QoL among Indian patients receiving haemodialysis and their spouses found that over half the patients were depressed and 42.8% of spouses were also depressed. Depressed spouses had significantly higher Revised Dyadic Adjustment Scale (RDAS) scores, poorer QoL, and more marital stress compared with non-depressed spouses [36]. A further study involving patients undergoing haemodialysis and their spouses (*n* = 38) and healthy controls (*n* = 38) assessed social support, stress, family functioning and marital satisfaction and quality [33]. There was a significant difference in stress reactions and social support in the three groups. Patients receiving haemodialysis treatment and their spouses had higher scores in stress reactions than the control group (*p* < 0.01, *p* < 0.05). Stress was negatively associated with marital satisfaction across the three groups (*p* < 0.001) [33].

## 4. Discussion

This review has synthesised existing published literature on the experiences, knowledge requirements and needs of informal caregivers of patients with ESKD on haemodialysis. These informal caregivers have significant unmet needs regarding carer information which has a negative impact on their overall well-being and their ability to provide effective care. Given the crucial importance and contribution of informal caregivers to their patients and to health services more needs to be done to support informal caregivers [42]. Therefore policies, legislation, professional guidance, and research all emphasise the case for identifying carers and addressing their needs [43,44,45,46,47]. 

The review highlights the difficulties informal caregivers experience in obtaining information from healthcare professionals, and in cases where information was obtained from healthcare professionals it was minimal. This finding was also reported in a previous review which focused on the needs of informal caregivers of patients with advanced cancer [48] where carers experienced difficulties obtaining sufficient information from healthcare professionals. Despite the growing recognition of the burden that informal caregivers of patients with ESKD receiving haemodialysis face, there is a lack of tailored resources/guidance to assist them in their caring role. This may be due to inadequate advocacy or a lack of funding and support resources available to develop and implement such resources/guidance. Moreover, in the clinical setting the main priority is responding to the needs of patients undergoing haemodialysis, with much less emphasis placed on supporting and educating their informal caregivers [49]. This contrasts with other chronic diseases such as stroke [50], cancer [51], and dementia [52] where personalised holistic and multicomponent caregiver support programmes have been developed to support informal caregivers and address their unmet needs. The Melbourne Family Support Programme is an example of one such intervention used in a palliative care setting, which used a psychoeducational intervention to reduce the burden of family caregivers. This programme produced significantly improved outcomes in family caregivers’ preparedness, competence, positive emotions, levels of psychological wellbeing, and unmet needs [53]. Support programmes akin to the Melbourne Family Support Programme should be developed and implemented as they could potentially improve quality of life, satisfaction, and ability to cope for those informal carers who provide care to patients with ESKD receiving haemodialysis.

This review identified high levels of caregiver burden and associated psychosocial distress, a finding which is consistent with studies that have investigated the care burden in caregivers of patients undergoing haemodialysis [54,55]. However, in other chronic diseases such as cancer and heart failure, the provision of carer-centred guidance significantly improved informal caregivers’ ability to cope with the challenges of their caring role, leading to a subsequent reduction in caregiver burden [56]. In addition, the current review reported a higher level of informal caregiver burden in caregivers who had to assist patients with personal care needs and those patients on dialysis who suffered other chronic diseases. Ageing, frailty, and multi-morbidity are highly prevalent in ESKD [57,58] placing significant burdens on informal caregivers highlighting the urgent need for the development of supportive interventions. 

Although there are few educational interventions for informal caregivers of patients undergoing haemodialysis, they were identified as being effective in enhancing informal caregiver skills [29,35]. Teaching coping strategies such as communication skills, anger management skills, and deep breathing for relaxation has been shown to be successful for informal caregivers of patients receiving haemodialysis and peritoneal dialysis to reduce caregiver burden and increase quality of life [29]. These findings are reflective of other chronic disease populations whereby informal caregivers are able to deal more confidently with the challenges of their caregiving role [59]. Specifically, problem-focused coping strategies have been shown to help in relation to interventions for caregivers of patients with dementia, heart failure, and cancer and have proven to be very effective in reducing caregiver burden [60,61,62]. Given the limited number of studies examining the effects of educational interventions for caregivers of patients with ESKD on haemodialysis, future research is needed with this cohort of informal caregivers to develop and test supportive interventions to increase generalisability and identify effectiveness over a longer period.

Research on informal caregivers of patients undergoing haemodialysis has mainly focused on the negative aspects of providing care (stress, depression, loss of earnings, and reduced quality of life), whereas this review highlights the importance of learning about caregivers who cope well (e.g., through peer support) to facilitate similar experiences. Informal caregivers’ emerging confidence and strength in coping with the burden of caregiving has been identified as resilience, namely individuals responding in a positive manner to a challenging situation [63]. The concept of resilience has been examined in informal caregivers of patients with a diagnosis of dementia. Caregivers who maintain a resilient mindset experienced their caring situation less negatively, coped better and maintained adaptive functioning, thus promoting more effective care [64,65]. Other factors such as perceived social support has been shown to mediate the association between resilience and caregiver burden among caregivers of older adults [66]. A recent review, which employed a peer-led-web-based resource to support informal caregivers of patients with cancer, highlighted the benefits of peer-led videos which allowed informal caregivers to hear the experiences of other caregivers which in turn helped to allay any feelings of helplessness and uncertainty experienced in their caregiving role [67,68,69,70]. 

### 4.1. Strengths and Limitations 

The strength of this narrative review is its description of the phenomenon of caregiving for a person with ESKD undergoing haemodialysis, through the views of informal caregivers, encompassing both positive and negative experiences. The use of CASP for quality appraisal has highlighted most of the included studies were of a low-quality rating limiting the conclusions drawn from the evidence. This has, however, highlighted the need for future robust research in this area. The inclusion of both qualitative and quantitative studies provided more informative findings on the experiences and unmet needs of informal caregivers in this cohort of patients. 

### 4.2. Implications for Clinical Practice 

Informal caregivers are pivotal in supporting and caring for patients with ESKD receiving haemodialysis. However, they describe a broad range of negative experiences and unmet needs associated with their caregiving role. Efforts to prepare informal caregivers to undertake their caring role should become a priority for healthcare professionals to ensure the early identification of need and support for informal caregivers, thus helping to maintain health and well-being of informal caregivers and the persons for whom they care. We can improve caregivers’ worries, problems, and treatments through the early identification of issues and concerns expressed by this cohort of informal caregivers resulting from their caregiving experiences [9,10]. For this to be effective, there needs to be open communication between the healthcare professional and the informal caregiver to support informal caregivers, to build a therapeutic relationship, and to foster a positive engagement [9]. The work currently being undertaken by the author aims to identify the needs and experiences of informal carers, with the aim of developing a tailored supportive intervention which is holistic and addresses the informal caregivers’ specific and ever-changing needs [11]. This will enable informal caregivers to gain knowledge and information, and to develop skills to allow them to deal more confidently and proficiently in their role as informal caregivers. 

### 4.3. Implications for Research 

The findings of this review have important implications to help identify core components of a supportive intervention which would comprehensively address informal caregivers’ individual needs based on their caregiving experiences [21]. This narrative review demonstrates there is a need for more rigorous qualitative studies that explore the perceived positive and negative aspects of informal caregiving and information needs of informal caregivers, especially given the increasing number of frail patients with multi-morbidity receiving haemodialysis. This underscores the importance of co-designing supportive interventions with informal caregivers and relevant stakeholders thus leading to the development of an intervention which is renal carer specific which responds to their individual needs. Without the valuable contribution of informal caregivers, the NHS would be under even greater strain [49]. The care which informal caregivers provide in the UK for example is estimated to be worth 132 billion GBP a year [71]. 

## 5. Conclusions

This review of the literature has highlighted that there are several unmet needs in the current research around the knowledge and informational needs and skills required by informal carers, thus necessitating the development of a supportive intervention to empower them in their caring role. Two studies showed that educational interventions are effective in enhancing caregiver skills since they reduce caregiver burden, enabling informal carers to deal more effectively with the demands and challenges of their caring role. However, there were no qualitative aspects to these studies, so there is a poor understanding of the personal experiences of this group of informal carers who have unmet needs [14]. Further research is required to better understand the everyday experiences of informal carers to identify what would assist and benefit them in their caregiving role and to explore the impact of educational interventions at different time points to differentiate between transitory and prolonged effects of these interventions. 

## Figures and Tables

**Table 1:** Search strategy used for Medline database.

Title Search Strategy Used for Medline Database
1. dialysis patients.mp. 2. hemodialysis patients.mp. 3. haemodialysis patients.mp. 4. hemodialysis.mp or exp Hemodialysis/5. haemodialysis.mp. 6. dialysis.mp. or exp Dialysis/7. 1 or 2 or 3 or 4 or 5 or 6
8. informal carers.mp. 9. (carer or carers).mp. 10. extended family.mp. or exp Extended Family/11. family member.mp. or exp Family Members/12. mothers.mp. or exp Mothers/13. fathers.mp. or exp Fathers/14. siblings.mp. or exp Siblings 15. spouses.mp. or exp Spouses/16. husband.mp. or exp Husbands/17. wife.mp. or exp Wives/18. caregivers.mp. or exp Caregivers/19. significant other.mp or exp Significant Others/20. 8 or 10 or 11 or 12 or 13 or 14 or 15 or 16 or 17 or 18 or 19
21. caregiver burden.mp. or exp Caregiver Burden/22. caregiver attitudes.mp. 23. attitude.mp 24. emotions.mp. or exp Emotions/25. thoughts.mp. 26. belief *.mp 27. Fee *.mp. 28. experience *.mp. 29. perception.mp. or exp Perception/30. view *.mp. 31. stress.mp. or exp Stress/32. psychological stress.mp. or exp Psychological Stress/33. personal satisfaction.mp. 34. 21 or 22 or 23 or 24 or 25 or 26 or 27 or 28 or 29 or 30 or 31 or 32 or 33
35. 7 and 20 and 34

* attached to the stem of a word allows searches for any word which includes that stem or the letters before the asterisk.

## Data Availability

Not applicable.

## Supplementary Materials

The following are available online at https://www.mdpi.com/article/10.3390/healthcare10010057/s1. The data extraction table (Table S1) of studies included in the narrative reviews has been included as a supplementary file.
