# Supplementary material for: Knowledge Requirements and Unmet Needs of Informal Caregivers of Patients with End-Stage Kidney Disease (ESKD) Receiving Haemodialysis: A Narrative Review
Source: Healthcare (Basel). 2021 Dec 29;10(1):57. doi: 10.3390/healthcare10010057 (PMC8775298; doi:10.3390/healthcare10010057)
Supplement: Supplementary file 1 [file healthcare-10-00057-s001.zip › healthcare-1489812-supplementary.pdf]

healthcare

MDPI

Table S1. Data extraction table of studies included in review.

Author(Year) and Country	Aim of Study	CASP Rating	Study Population	Study Design	Key Findings	Limitations
Alnazly (2016) Jordan.	Burden and Coping Strategies among Jordanian Caregivers of Patients undergoing Haemodialysis.	Low	139 Informal caregivers over three dialysis units in Jordan.	Questionnaire study.	• The Oberst Caregiving Burden Scale (OCBS) was significantly related to self-controlling (r = 0.20) and seeking social support (r = 0.17).<br>• Caregiver burden was positively and significantly correlated with self-controlling coping subscale ($p = 0.05$).<br>• Living with the patient was the only significant predictor of burden ($p = 0.00$).	• It is unclear from the information provided relating to data collection if/or what information caregivers were provided with prior to completing the questionnaires.<br>• It is not clear from the description given who recruited the caregivers to the study.<br>• No sample size calculation was carried out to determine statistical power.<br>• Caregivers had to be aged at least 21 years to be included in the study.
Alnazly (2018) Jordan.	Impact of an educational intervention in care-giving outcomes in Jordanian caregivers of patients receiving haemodialysis.	Low.	169 informal caregivers of patients receiving haemodialysis.	One group pre-test post-test study.	• Caregivers were moderately burdened, and their lives changed for the worse because of caregiving.<br>• Mean scores measuring caregiver burden was lower prior to initiating the intervention as compared to the post intervention scores.<br>• The post intervention mean caregiver burden scores indicate that the information and educational intervention had a	• Effects of the intervention were tested immediately post-intervention; the study therefore is limited to the short-term impacts of the intervention.<br>• The one-group pre-test post-test study design poses a threat to internal validity.<br>• A pre-test post-test design may not allow for accurate assessment of the program's effectiveness as it lacks a control group for comparison.

					positive influence on caregiving outcomes ($p < 0.05$).	
Barnieh et al (2014)<br><br>Canada.	Views of Canadian patients on or nearing dialysis and their caregivers.	Low	189 informal caregivers of patients on or nearing dialysis.	Secondary analysis of a survey.	• Carers expressed the need for more information and guidance to assist in their caring role.<br>• Carers needed more information about the pathology of ESKD, haemodialysis treatment, complications, dietary needs, and medications.<br>• Lack of knowledge and information impacted carer's ability to provide effective care.<br>• Information provided was non-specific to haemodialysis.<br>• Timing of information was a concern of carers as was the need for ongoing information sources.<br>• Informal carers identified peer support as a valuable resource.	• Qualitative analysis of survey did not allow follow up questions.<br>• Analysing written responses, without further clarification from respondents could result in misclassification of certain statements.<br>• Some responses were not included in the data analysis as the researcher felt they were not informative in the context of aim of this study.
Ebadi et al (2018)<br><br>Iran.	Study on Personal Life among Family Caregivers of Haemodialysis Patients.	Low.	19 informal caregivers of patients undergoing haemodialysis.	Semi-structured interviews.	• Informal carers felt overwhelmed due to the many roles in their own lives in addition to the responsibilities of informal care.<br>• Conflict between caring role and that of parental and spousal responsibilities.<br>• Responsibilities of caring and patients' unstable condition resulted in carers not able to	• Little detail provided into semi-structured interview schedule.<br>• Researchers reported they tried to increase the ability to generalise concerning maximum variability in the selection of participants. No information however is given about variability of participants.

					spend their leisure time as they would like.<br>• Conflict between caring and occupational roles such as delays in performing assigned tasks, reduced mental concentration led to dissatisfaction with line managers.	
Eslami et al (2016)<br><br>Iran.	To investigate the coping skills of Iranian family caregivers in caring for patients undergoing haemodialysis.	Low.	20 informal family caregivers.	In-depth and unstructured interviews.	• Gathering information and continuous learning about patients care-related needs was a necessary skill in achieving adaptability.<br>• Caregivers used self-nurturing skills to help deal with the problems arising from their caring role.<br>• Caregivers never neglected themselves - paying attention to oneself led to more efficacious caring.<br>• Caregivers sought to renew their own spirit by turning to programs of recreation and spirit renewal to manage problems resulting from their caring role.	• Participants were enrolled from public health centres in Iran, therefore the transferability of the findings are limited.<br>• The lack of detail and ambiguity surrounding the responses reported restricts the inferences which can be drawn.
Eslami et al (2018)<br><br>Iran.	To identify perspectives and experiences of Iranian family caregivers of haemodialysis patients.	Low.	25 family caregivers of patients undergoing haemodialysis	Qualitative study using in-depth and unstructured interviews and field observation.	• Changing nature of the patients' needs led to a decline in the ability of carers to provide care.<br>• Negative effects on caregivers' daily activities, occupation, exercise, and recreation.	• Study involved 25 family caregivers; however, data collection reports 34 interviews were completed.<br>• There is lack of clarity around whether there was more

					• As the patient's disease intensified, coping with and adaptability to new conditions became more difficult.<br>• Trying to deal with patient's problems often led to feelings of hopelessness by the carer.	than one haemodialysis unit included in the study.<br>• There is a minimum amount of qualitative data presented.
Ghane et al (2016)<br><br>Iran.	To determine the effectiveness of problem-focused coping strategies on the burden on caregivers of haemodialysis patients.	Moderate	76 family caregivers of haemodialysis patients.	Randomised controlled trial.<br><br>Intervention group (n = 38) received four training sessions on problem focused coping strategies.<br><br>Control group (n = 38) received no intervention.	• No significant difference between the baseline mean caregivers' burden scores of the intervention and control groups (p = 0.308).<br>• Mean caregiver burden in the intervention group decreased, with a significant difference between both groups at the end of the study (p < 0.001).	• The small sample size and relatively short follow up period limit generalisability of findings.<br>• Lack of longitudinal follow up fails to show any long-term improvement.<br>• Small sample size (but adequately powered).<br>• Well described and conducted study.
Gray et al (2019)<br><br>Australia.	Quality of life in caregivers compared with dialysis recipients: The Co-Active substudy of the ACTIVE dialysis trial.	Low.	54 patient caregiver dyads.	Prospective observational cohort study.	• University education predicted higher caregiver MCS (10.3 (95% CI: 4.2-16.3), p < 0.001) and PCS (7.5 (95%CI: 1.7-13.2), (p = 0.011).<br>• Caregiver health utility was higher in those with university education (p = 0.001).	• Study results primarily applicable to caregiver's resident in China.<br>• Dialysis patients cared for in this study were generally younger with less co-morbidities than is typical in the haemodialysis population.<br>• Questionnaires were completed at baseline and follow up. No time frame is provided for follow up, and the baseline results are only reported.<br>• Inconsistencies surrounding the data collections tools

						used for Chinese and non-Chinese speaking caregivers, with no explanation given.
Hoang et al (2019)<br><br>Vietnam.	To determine associations between activities and burden in Informal carers of people undergoing haemodialysis.	Moderate	178 informal caregivers of patients undergoing haemodialysis.	Cross sectional study.	• Carers reported a high level of caregiver burden with 80.9% identifying the level as severe.<br>• Predictors of greater informal caregiver burden were having own health problems, greater time required to provide care, and doing more difficult tasks ($p < 0.01$).<br>• Caregivers who provided more than 20 hours of care per week or had been a caregiver for more than five years reported significantly higher burden ($p < 0.05$).	• Convenience sampling from a single haemodialysis centre limits generalisability of results.<br>• The cross-sectional study design does not allow any casual inferences to be made.
Jafari et al (2018)<br><br>Iran.	To examine the relationship between care burden and quality of life in caregivers of haemodialysis patients.		246 caregivers of haemodialysis patients.	Descriptive-analytical study.	• 37.4% of caregivers experienced high or very high levels of care burden.<br>• 42.7% experienced a moderate level of care burden.<br>• Significant and negative correlation between total scores of care burden and quality of life ($p < 0.001$).<br>• Factors influencing care burden include level of patient's caring capability, incidence of other chronic diseases.<br>• Significant relationship between level of caregivers'	• Impossibility of determining the relationship between cause and effect due to the diversity of the study.<br>• Data collection method based on self-reporting so reliability and validity of the findings could be questioned as no objective measures were used.<br>• The lack of a control group limits the ability to establish a cause-and-effect relationship.<br>•

					education and care burden ($p <$ 0.001), increased education level decreased care burden.<br>• Positive correlation between patient's incident of other chronic diseases and burden of caregivers ($p < 0.001$), Caregiver burden was significantly higher when caring for a patient with other chronic diseases, compared with carers caring for a patient without another chronic disease.	
Jiang et al (2015) China.	Family functioning, martial satisfaction and social support in haemodialysis patients and their spouses.	Low.	38 patients and their spouses and 38 healthy controls.	Quantitative study.	• Patients receiving haemodialysis treatment and their spouses had higher scores in stress reactions than the control group ($p < 0.01$, $p < 0.05$).<br>• Stress was negatively associated with marital satisfaction across the three groups ($p <$ 0.001).<br>• Support outside family was positively related to marital quality and satisfaction in healthy individuals ($p < 0.001$) and negatively related to marital quality in patients ($p < 0.001$) and their spouses ($p < 0.001$).<br>• Support within family was associated positively with family cohesion and adaptability among patients and healthy	• Self-reported measures and relatively small sample size.<br>• Patients included in this study received haemodialysis twice weekly only, in UK treatment is routinely delivered three times weekly.<br>• Patients were required to be aged over 22 years to participate, which could affect generalisability of findings. In the UK people attain majority of age at 18 years.<br>The exclusion criteria were very stringent which is likely to impact how representative participants are in relation to the general haemodialysis population.

					individuals ($p < 0.001$) but not among spouses	
Khaira et al (2012)<br><br>India.	Depression and Marital Dissatisfaction among Indian Haemodialysis Patients and their Spouses.	Low.	49 (32 males and 17 females) patients on haemodialysis and their spouses.	Cross-sectional Study.	• Patients living in extended families had lower BDI scores, higher QOL, and lower marital stress ($p < 0.01$) compared with those living in nuclear families.<br>• Depressed spouses had significantly higher RDAS scores and poorer QOL compared with nondepressed spouses.<br>• Depression in patients had a direct correlation with depression in spouse and degree of marital dissatisfaction in spouse ($p < 0.0001$).<br>•	• Cross-sectional study design and small participant numbers limits the establishment of cause-effect relationships.<br>• Results are not representative of patients receiving haemodialysis in India as this dialysis unit is transplant-orientated, patients are younger and have shorter duration on haemodialysis before receiving a transplant.<br>• In addition to questionnaires face-to-face interviews were completed, but no reference was made to the content of the interviews or findings. Extensive exclusion criteria which could have introduce sampling bias and limited the potential sample population.
Kilie and Kaptanogullari (2017)<br><br>Cyprus and Turkey.	To evaluate the burden of Caregivers of patients undergoing haemodialysis in two different communities – Northern Cyprus and Istanbul.	Low	210 family caregivers of patients undergoing haemodialysis.	Descriptive Study.	• Caregiver burden scale scores showed a significant difference according to the treatment centre ($p < 0.000$).<br>• Mean caregiver burden was higher in carers living in Cyprus compared to those living in Turkey.<br>• The combined effect of treatment centre and educational status of the caregiver on the burden scale score was not	• The nuclear family structure predominates in Northern Cyprus, whereas traditional family structure predominates in Istanbul. Caregivers in Istanbul, therefore are more likely to receive support from relatives, friends and social support. These cultural characteristics will influence study findings.

					statistically significant (*p* = 0.724), whereas the relation of the caregiver to the patient significantly affected the caregiver burden scale score of the caregiver (*p* < 0.008).	• No sample size calculation was completed to determine number of caregivers required.
Lima et al (2017)<br><br>Portugal.	Family Perceptions of Care with Patients in Renal Dialysis	Low	Eight family caregivers.	Semi-structured interviews.	• When necessary knowledge was provided informal carers were comfortably engaged in their caring role.<br>• Lack of knowledge left carers unsure how to fulfil their caring role and increased anxiety and stress.<br>• Carers needed knowledge of pathology of ESKD, potential complications of the disease, medications, and dietary and fluid requirements.<br>• Need for good communication between carers and health professionals.<br>• Carers had difficulty in obtaining information form health professionals.	• Conducted in one haemodialysis setting in Brazil.<br>• No insight provided into the topic guide for the semi-structured interviews.<br>• Methodology poorly described.<br>• All caregivers identified from the Family Health Strategy which is unique to Brazil.<br>• Small sample size.
Rabiei et al (2016)<br><br>Iran.	Caring in an atmosphere of uncertainty: perspectives and experiences of caregivers of people undergoing haemodialysis in Iran.	Moderate.	20 haemodialysis family caregivers.	In-depth and unstructured interviews and field observation.	• Lack of individualised/tailored information.<br>• Informal carers needed knowledge regarding pathology of ESKD, haemodialysis treatment and complications and how to manage these, medication management, how to	• The generalisability of the data could be influenced as carers were selected from three public healthcare centres in Iran.<br>• Potential problem with reliability as every unstructured interview is unique as different questions may be asked to different carers depending on

					prepare meals within limitations of dietary restrictions.<br>• Focus by healthcare professionals on information for patients, not their carers. Any information provided by health workers for carers was usually passed via the patient and detail was not always relayed properly.<br>• Trying to obtain direct information from healthcare staff was a serious concern for carers.<br>• In absence of expertise or support systems carers often sought information sources which were not individualised to care needs of the patient they were caring for, leading to confusion.<br>• Experiential learning led to carers facing a plethora of information which focused on general care, non-specific to the care of renal patients.<br>• Information between health workers and carers should be tailored to the stage of the disease.	responses, therefore making the study difficult to replicate.
Rafati et al (2019)<br><br>Iran.	Caregiver Burden and Spiritual Well-being in Caregivers of Haemodialysis Patients.	Low.	382 informal caregivers of patients receiving haemodialysis.	Correlational study across six dialysis units.	• Significant relationship between the mean score of caregivers with the patients' ability to perform daily activities ($p < 0.001$) indicating the less ability	• Findings not generalisable as based in haemodialysis settings in Iran.<br>• Methods section poorly described.

					to perform personal activities the higher the caregiving burden.<br>• Caregivers with lower than diploma education had a higher burden than caregivers with diploma and higher diploma, unemployed caregivers had a higher burden score than employed caregivers ($p < 0.001$) and housewife caregivers ($p = 0.049$).<br>• There was a significant relationship between caregiver burden scores and spiritual well-being scores ($p < 0.001$) indicating that finding satisfaction and peace with faith in God was a strong facilitator for the ability to care for haemodialysis patients.	• Data collection using questionnaires, cross-sectional study design and census sampling method.
Starks et al (2019)<br><br>United States of America.	Factors associated with Quality of Life of Family Caregivers of Dialysis Recipients.	Low.	75 African American female caregivers of patients receiving haemodialysis.	Secondary data analysis using a descriptive correlational design.	• Lower caregiver QOL scores were associated with a higher number of hours worked ($p < 0.001$) and higher levels of education ($p = .31$).<br>• Higher QOL was associated with living with the care recipient compared with caregivers who did not live with the care recipient.<br>• QOL scores were significantly associated with caregiver burden scores and family functioning scores. Caregivers with higher QOL reported lower caregiver burden scores ($p = 0.002$).	• Limitations are associated with conducting a secondary data analysis, namely the operational and theoretical definitions sample all of which were predetermined for other purposes.<br>• External validity of the study is limited to demographically similar caregivers recruited from the south-eastern United States.

					• Caregiver QOL was negatively associated with caregiver education, caregiver employment status, and caregiver burden.	
Turner and Finch-Guthrie (2020)<br><br>United States of America.	Experiences of Caregivers for a Family Member who is Using Haemodialysis.	Moderate.	Six family caregivers of inpatients from a medical-renal nursing unit.	Qualitative phenomenological study	• Alongside the physical requirements of caregiving, carers report much of the work is cognitive in nature, such as making medical appointments, arranging medications and meal planning.<br>• Caregivers in employment needed the support of their manager in addition to that of their work colleagues to schedule time for caring duties which needed to be carried out during working hours.<br>• When caregivers were unable to do special things for themselves like having lunch with friends or going to the movies it added to the feeling of being overwhelmed.<br>• Stress was recognised as part of the caregiving experience, which manifested in anger or frustration and was often reported as a decline in the health status of the caregiver.<br>• The stress of informal caring often led to poor coping	• Small sample size (however researcher believes data saturation was reached as no new information was forthcoming after the fifth interview).<br>• Interviews were completed with caregivers when the patients they were caring for where in hospital, which may have influenced responses.<br>• Study focused on caregivers in one geographical area therefore limiting the generalisability of the findings.<br>• Study sample consisted of homogeneous caregivers who spoke, read and wrote in English, the perceptions and meaning of caregiving in non-English speaking caregivers and those from other cultures may be different.

	mechanisms such as smoking and eating junk food.<br>• Caregivers reported anger and frustration with their caring situation. For some, caregivers the anger is directed towards the family member for not following physician orders when they were younger to avoid the sequalae of chronic disease.<br>• Restrictions in lifestyle, social routines, travel, and the ability to participate in recreational activities are associated with caregiving.	
